# Supplementary material for: Resolving the full spectrum of human genome variation using Linked-Reads
Source: Genome Res. 2019 Apr;29(4):635–45. doi: 10.1101/gr.234443.118 (PMC6442396; doi:10.1101/gr.234443.118)
Supplement: Supplemental Material [file supp_gr.234443.118_Supplemental_longranger-2.2.zip › longranger-2.2.tar.bz2!/lr2.2-public/lib/stan/cmdstan/stan/doc/api/html/structstan_1_1gm_1_1is__no__op__statement__vis.html]

Stan: stan::gm::is\_no\_op\_statement\_vis Struct Reference


|  |  |
| --- | --- |
|  | Stan  2.5.0  probability, sampling & optimization |


- Main Page
- Related Pages
- Namespaces
- Classes
- Files

- Class List
- Class Index
- Class Hierarchy
- Class Members

All Classes Namespaces Files Functions Variables Typedefs Enumerations Enumerator Friends Macros Pages

- stan
- gm
- is\_no\_op\_statement\_vis

Public Member Functions |
List of all members

stan::gm::is\_no\_op\_statement\_vis Struct Reference

`#include <ast.hpp>`

Inheritance diagram for stan::gm::is\_no\_op\_statement\_vis:

|  |  |
| --- | --- |
| Public Member Functions | |
| bool | operator() (const nil &st) const |
|  | |
| bool | operator() (const assignment &st) const |
|  | |
| bool | operator() (const sample &st) const |
|  | |
| bool | operator() (const increment\_log\_prob\_statement &t) const |
|  | |
| bool | operator() (const expression &st) const |
|  | |
| bool | operator() (const statements &st) const |
|  | |
| bool | operator() (const for\_statement &st) const |
|  | |
| bool | operator() (const conditional\_statement &st) const |
|  | |
| bool | operator() (const while\_statement &st) const |
|  | |
| bool | operator() (const print\_statement &st) const |
|  | |
| bool | operator() (const reject\_statement &st) const |
|  | |
| bool | operator() (const no\_op\_statement &st) const |
|  | |
| bool | operator() (const return\_statement &st) const |
|  | |

## Detailed Description

Definition at line 671 of file ast.hpp.

## Member Function Documentation

|  |  |  |  |  |  |
| --- | --- | --- | --- | --- | --- |
| bool stan::gm::is\_no\_op\_statement\_vis::operator() | ( | const nil & | *st* | ) | const |

Definition at line 1246 of file ast\_def.cpp.

|  |  |  |  |  |  |
| --- | --- | --- | --- | --- | --- |
| bool stan::gm::is\_no\_op\_statement\_vis::operator() | ( | const assignment & | *st* | ) | const |

Definition at line 1249 of file ast\_def.cpp.

|  |  |  |  |  |  |
| --- | --- | --- | --- | --- | --- |
| bool stan::gm::is\_no\_op\_statement\_vis::operator() | ( | const sample & | *st* | ) | const |

Definition at line 1252 of file ast\_def.cpp.

|  |  |  |  |  |  |
| --- | --- | --- | --- | --- | --- |
| bool stan::gm::is\_no\_op\_statement\_vis::operator() | ( | const increment\_log\_prob\_statement & | *t* | ) | const |

Definition at line 1255 of file ast\_def.cpp.

|  |  |  |  |  |  |
| --- | --- | --- | --- | --- | --- |
| bool stan::gm::is\_no\_op\_statement\_vis::operator() | ( | const expression & | *st* | ) | const |

Definition at line 1258 of file ast\_def.cpp.

|  |  |  |  |  |  |
| --- | --- | --- | --- | --- | --- |
| bool stan::gm::is\_no\_op\_statement\_vis::operator() | ( | const statements & | *st* | ) | const |

Definition at line 1261 of file ast\_def.cpp.

|  |  |  |  |  |  |
| --- | --- | --- | --- | --- | --- |
| bool stan::gm::is\_no\_op\_statement\_vis::operator() | ( | const for\_statement & | *st* | ) | const |

Definition at line 1264 of file ast\_def.cpp.

|  |  |  |  |  |  |
| --- | --- | --- | --- | --- | --- |
| bool stan::gm::is\_no\_op\_statement\_vis::operator() | ( | const conditional\_statement & | *st* | ) | const |

Definition at line 1267 of file ast\_def.cpp.

|  |  |  |  |  |  |
| --- | --- | --- | --- | --- | --- |
| bool stan::gm::is\_no\_op\_statement\_vis::operator() | ( | const while\_statement & | *st* | ) | const |

Definition at line 1270 of file ast\_def.cpp.

|  |  |  |  |  |  |
| --- | --- | --- | --- | --- | --- |
| bool stan::gm::is\_no\_op\_statement\_vis::operator() | ( | const print\_statement & | *st* | ) | const |

Definition at line 1273 of file ast\_def.cpp.

|  |  |  |  |  |  |
| --- | --- | --- | --- | --- | --- |
| bool stan::gm::is\_no\_op\_statement\_vis::operator() | ( | const reject\_statement & | *st* | ) | const |

Definition at line 1276 of file ast\_def.cpp.

|  |  |  |  |  |  |
| --- | --- | --- | --- | --- | --- |
| bool stan::gm::is\_no\_op\_statement\_vis::operator() | ( | const no\_op\_statement & | *st* | ) | const |

Definition at line 1279 of file ast\_def.cpp.

|  |  |  |  |  |  |
| --- | --- | --- | --- | --- | --- |
| bool stan::gm::is\_no\_op\_statement\_vis::operator() | ( | const return\_statement & | *st* | ) | const |

Definition at line 1282 of file ast\_def.cpp.

---

The documentation for this struct was generated from the following files:

- src/stan/gm/ast.hpp
- src/stan/gm/ast\_def.cpp


---

[ Stan Home Page ]

*© 2011–2014,
Stan Development Team.*
